# Retrospective accuracy analysis of major guidelines and factors for lymph node metastasis of pT1 colorectal cancer

**DOI:** 10.1055/a-2697-7786

**Published:** 2025-10-09

**Authors:** ChunChi Lin, Wen-Yih Liang, Jui-Shen Chang, Shih-Ching Chang, Shung-Haur Yang

**Affiliations:** 1 46615 Division of Colon and Rectal Surgery, Department of Surgery, Taipei Veterans General Hospital, Taipei City, Taiwan; 2 210821 Department of Surgery, School of Medicine, National Yang Ming Chiao Tung University, Taipei City, Taiwan; 3 46615 Department of Pathology and Laboratory Medicine, Taipei Veterans General Hospital, Taipei City, Taiwan; 4 210821 Department of Pathology, School of Medicine, National Yang Ming Chiao Tung University, Taipei, Taiwan; 5 38016 Department of Surgery, En Chu Kong Hospital of the Hsing Tian Kong Foundation Medical Mission, New Taipei City, Taiwan

**Keywords:** Endoscopy Lower GI Tract, Colorectal cancer, GI Pathology, GI surgery

## Abstract

**Background and study aims:**

The aim of this study was to validation the accuracy of major guidelines and their adopted unfavorable histologic factors for lymph node metastasis (LNM) in pT1 colorectal cancer (CRC). National Comprehensive Cancer Network (NCCN), Japanese Society for Cancer of the Colon and Rectum (JSCCR), European Society for Medical Oncology (ESMO), and French Intergroup Clinical Practice Guidelines (FICPG) were included.

**Patients and methods:**

Retrospectively, 519 cases receiving oncological resection with lymphadenectomy were included. Factors and four pathological parameters--histological differentiation grades (HDG), lymphovascular invasion (LVI), depth of submucosal invasion (DSI)(>1000μm), and tumor budding (TB)--were analyzed for their impact on LNM incidence individually and in combination as defined by the guidelines.

**Results:**

HDG, LVI, TB, and gender (female) are risk factors for LNM in multivariate analysis. All guidelines have significant ability to predict LNM ( *P* <0.001). NCCN and ESMO have similar performance, in terms of sensitivity (63.8%/68.1%) and specificity (62.7%/69.5%). JSCCR and FICPG had similar performance in terms of good sensitivity (100%/100%) and low specificity (25.6%/25.2%). The JSCCR/FICPG group had higher sensitivity and lower specificity and accuracy than the NCCN/ESMO group.

**Conclusions:**

HDG, LVI, TB, and gender (female) are independent risk factors for LNM of T1 CRC. DSI is an excellent negative predictor, although not an independent risk factor. The NCCN/ESMO guideline has medium sensitivity and requires improvement. The JSCCR/FICPG guideline has perfect sensitivity but low specificity, thus exposing patients to many unnecessary surgeries. There is revision potential for current guideline factors, including those beyond current pathological ones, to improve LNM prediction accuracy.

## Introduction

With the introduction of population-based screening programs and advancements in modern clinical tools, the detection rate for early colorectal cancer (CRC) has been increasing in Taiwan 1 and worldwide 2 . As the incidence of pT1 CRC rises, necessity for standard radical resection of the affected colorectum after local excision (LE), including endoscopic or transanal, has been debated, given that the regional lymph node metastasis (LNM) rate in pT1 CRC is reported to be approximately 10% to 13% 3 4 . This suggests that surgical resection may not be required for the majority of pT1 CRC cases. Guidelines addressing LNM risk factors for pT1 CRC, including those from National Comprehensive Cancer Network (NCCN) Clinical Practice Guidelines in Oncology (NCCN Guidelines), Japanese Society for Cancer of the Colon and Rectum (JSCCR), European Society for Medical Oncology (ESMO), and French Intergroup Clinical Practice Guidelines (FICPG), primarily emphasize the histopathological characteristics of the primary tumor, with some variations ( Table 1 ) 4 5 6 7 8 .

**Table:** **Table 1** Unfavorable factors adopted by major guidelines (NCCN, JSCCR, ESMO, FICPG).

	**NCCN**	**JSCCR**	**ESMO**	**FICPG**	
				**Flat/sessile**	**Pedunculated**
Unfavorable risk factor	Margin (+) or fragmented	Margin (+)	LVI	Margin (+)	Margin (+)
	HDG 3 or 4	DSI (> 1000 μm)	HDG 3	HDG 3	HDG 3
	LVI or PNI	LVI	TB Grade 2/3	DSI (> 1000 μm)	Haggitt 4
	TB Grade 3	Histology	Pedunculated, Haggitt 4	LVI	LVI
		HDG 3 adenocarcinoma		TB Grade 2/3	TB Grade 2/3
		Signet ring cell carcinoma			
		Mucinous carcinoma			
		TB Grade 2/3			
DSI, deep submucosal invasion; ESMO, European Society for Medical Oncology; FICPG, French Intergroup Clinical Practice Guidelines; HDG, histology differentiated grade; JSCCR, Japanese Society for Cancer of the Colon and Rectum; LVI, lymphovascular invasion; NCCN, National Comprehensive Cancer Network; PNI, perineural invasion; TB, tumor budding.					

These guidelines adopt the concept of unfavorable risk factors to predict LNM, which subsequently inform recommendations for additional surgical resection. Among these guidelines, JSCCR includes the most comprehensive list of unfavorable risk factors: positive resection margins following LE, histological differentiation grade (HDG), lymphovascular invasion (LVI), depth of submucosal invasion (DSI) (> 1000 μm) 9 , and tumor budding (TB) 10 .

NCCN Guidelines do not explicitly include DSI as an unfavorable histologic feature, and TB criteria with only Grade 3 included is inconsistent with others 5 6 . Furthermore, NCCN has an indefinite algorithm regarding endoscopic classification of sessile lesion without risk factors. In ESMO Guidelines, DSI is defined as level 4 (Haggitt) invasion for pedunculated polyps, representing invasion deeper than the stalk 11 . However, for sessile or flat malignant polyps, ESMO does not clearly define DSI; any presence of other unfavorable histologic features would prompt a recommendation for adjuvant surgery 7 . Recently, FICPG made separate criteria for pedunculated and flat/sessile lesion 8 , aligning with ESMO for pedunculated lesions and with JSCCR for sessile/flat lesions.

Our study is based on the hypothesis that all pT1 CRC cases can be accurately identified, completely resected en bloc with clear margins using an endoluminal approach, adequately retrieved and fixed, and comprehensively evaluated by the pathologist. We hypothesize that LNM risk can be correlated with specific pathological parameters. Retrospectively, only cases that underwent oncological resection with lymphadenectomy were included for analysis. Pathological parameters, as defined by these guidelines, were analyzed for their individual and combined impacts on LNM incidence.

## Patients and methods

**Fig. 1:**
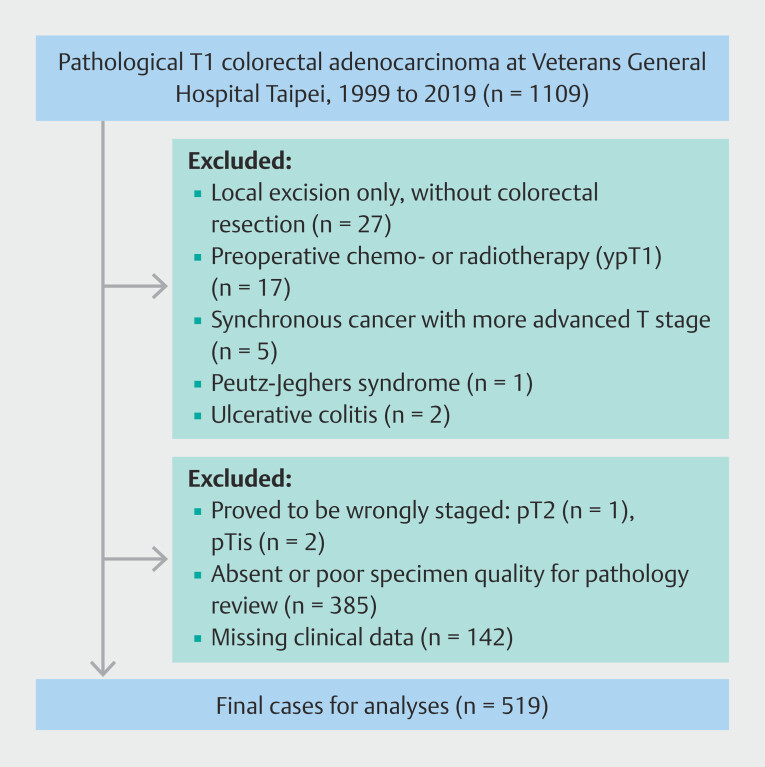
Flow chart of enrolled patients.

From 1999 to 2019, a total of 1,109 cases diagnosed with T1 CRC at Taipei Veterans General Hospital were retrospectively collected. Most patients with T1 CRCs would undergo colorectal resection once diagnosed during the study period. Only in recent years have JSCCR guidelines been adopted in our institution. Exclusion criteria included patients undergoing only local or endoscopic excision without colorectal resection, neoadjuvant chemotherapy or radiotherapy, synchronous CRC more advanced than the pT1 stage, familial adenomatous polyposis, Peutz-Jeghers syndrome, and ulcerative colitis ( Fig. 1 ). The majority of cases further excluded were due to absent or poor specimen quality for pathology review and missing clinical data.

On further examination, cases that were not confirmed as pT1 stage on review, those with absent or incomplete pathological materials, or cases with missing clinical data were excluded. Ultimately, 519 cases were included in the analysis. The study followed the TNM staging system of the American Joint Committee on Cancer (AJCC) (8th edition) 12 .

The study protocol was reviewed and approved by the Institutional Review Board of Taipei Veterans General Hospital (IRB-TPEVGH: 2022–09–004CC). Available pathological slides with hematoxylin and eosin staining (H&E stain) were retrieved, digitally scanned, and reviewed by a dedicated gastrointestinal pathologist (WY Liang). Four pathological parameters outlined in the guidelines were meticulously reviewed and recorded, including HDG defined by the World Health Organization (WHO) Classification of Digestive System Tumors (5 ^th^ ed) (2019) 13 , SMID defined by “Japanese Classification of Colorectal, Appendiceal, and Anal Carcinoma, third English edition” 9 , LVI defined as either carcinoma cells identified within endothelium-lined channels or tumor emboli identified within endothelium-lined channels surrounded by a smooth muscle wall 14 , and TB defined by International Tumor Budding Consensus Conference (ITBCC) 10 . The definition and evaluation method for HDG differs between JSCCR (predominant differentiation pattern) and NCCN/ESMO/FICPG (least differentiated component). Clinical information was reviewed and the endoscopic images were also reviewed and classified according to the Paris endoscopic classification of superficial neoplastic lesion 15 .

Statistical analyses were conducted using IBM SPSS Statistics version 25. Categorical variables were analyzed using the Chi-square test and Fisher’s exact test. Variables with *P <* 0.1 were included in the Multiple Cox Regression analysis (Backward Wald) to identify independent factors. *P* < 0.05 was considered statistically significant.

## Results

Table 2 summarizes clinicopathological characteristics. pN+ was found in 47 cases (9.1%), including one case of pN2, which was also stage IV; no cases of N1c were observed. The percentage of male patients was slightly higher (57%). The median number of lymph nodes (LN) harvested was 12 (interquartile range [IQR] 9–17). The carcinoembryonic antigen level before any LE, available for 503 cases, was a median of 2.14 ng/mL (IQR 1.6–3.0). In terms of endoscopic morphology, pure early polypoid lesions (type 0-I) made up 45.1%, including pedunculated (type 0-Ip) 5.2%, semi-pedunculated (type 0-Isp) 9.6%, and sessile (type 0-Is) 30.2%. Early non-polypoid and mixed types made up 54.9% of findings. Most histological types were adenocarcinoma (99%). There were also one case of mucinous adenocarcinoma, three signet ring cell carcinomas, and one medullary carcinoma. In HDG (WHO 5 ^th^ ), poorly differentiated (Gr3) comprises 27%. DSI was present in 76.1%, LVI in 6.6%, and TB Gr 2 (7.1%) and Gr 3 (12.9%) were observed. Among included cases, 98.7% (n = 512) was from cases undergoing primary colorectal resection, and only 1.3% (n = 7) was from receiving initial LE. It was because specimens of other cases receiving initial LE and following colorectal resection were absent or poor quality for pathology review, and therefore, were excluded. All parameters were analyzed for their association with LNM incidence. In univariate analysis, five risk factors—HDG, DSI, LVI, TB, and gender (female)—were significant for LNM and were included in further multivariate analysis. In multivariate analysis ( Table 3 ), HDG, LVI, TB, and gender (female) remained significant independent risk factors.

**Table:** **Table 2** Clinicopathological characteristics of pT1 Cases (n = 519).

		**Overall**	**LNM-**	**LNM+**	***P***
Gender, n (%)					
Male		296 (57%)	276 (53.2%)	20 (3.9%)	
Female		223 (43%)	196 (37.8%)	27 (5.2%)	0.035
Age, y, median (IQR) 65.8 (57.8–75.0)					
< 65.8		259 (49.9%)	233 (44.9%)	26 (5%)	
≥ 65.8		260 (50.1%)	239 (46.1%)	21 (4%)	0.44
Polyp size, mm, median (IQR) 20 (15–30)					
< 20		280 (53.9%)	252 (48.6%)	28 (5.4%)	
≥ 20		239 (46.1%)	220 (42.4%)	19 (3.7%)	0.42
CEA, ng/mL, median (IQR) (n = 503) 2.14 (1.6–3.0)					
< 5.0		471 (93.6%)	429 (85.3%)	42 (8.3%)	
≥ 5.0		32 (6.4%)	28 (5.6%)	4 (0.8%)	0.49
Lymph nodes totally retrieved number, median (IQR)		12 (9–17)			
< 12		184 (35.3%)	229 (44.1%)	29 (5.6%)	
≥ 12		335 (64.5%)	303 (90.4%)	32 (9.6%)	0.59
Tumor location					
Right colon		139 (28.7%)	139 (26.8%)	10 (1.9%)	
Left colon		173 (36.8%)	174 (33.5%)	17 (3.3)	
Rectum		159 (34.5%)	159 (30.6%)	20 (3.9%)	0.37
Endoscopic morphology type					
0-Ip	Pedunculated	27 (5.2%)	26 (5%)	1 (0.2%)	
0-Isp	Semi-pedunculated	50 (9.6%)	48 (9.2%)	2 (0.4%)	
0-Is	Sessile	157 (30.2%)	142 (27.4%)	15 (2.9)	
Other or mixed types		285 (54.9%)	256 (49.3%)	29 (5.6%)	0.39
N Category, AJCC					
N0		472 (90.9%)	472 (90.9%)	0	
N1		46 (8.9%)	0	46 (8.9%)	
N2		1 (0.2%)	0	1 (0.2%)	< 0.001
Stage, AJCC					
Stage I		472 (90.9%)	472 (90.9%)	0	
Stage III		46 (8.9%)	0	46 (8.9%)	
Stage IV		1 (0.2%)	0	1 (0.2%)	< 0.001
Histology type					
Adenocarcinoma		514 (99%)	468 (90.2%)	46 (8.9%)	
Mucinous adenocarcinoma		1 (0.2%)	1 (0.2%)	0	
Signet ring cell carcinoma		3 (0.6%)	2 (0.4)	1 (0.2%)	
Medullary carcinoma		1 (0.2%)	1 (0.9%)	0	0.5
Histologic grade (Gr) (WHO 5th)					
Gr1/Gr2, well/moderately differentiated		379 (73%)	355 (68.4%)	24 (4.6%)	
Gr3, poorly differentiated		140 (27%)	177 (22.5%)	23 (4.4%)	< 0.001
Main histology (JSCCR)					
Tubular/papillary		507 (99.7%)	462 (89%)	45 (8.7%)	
Poorly differentiated/mucinous/signet ring cell		12 (2.3%)	10 (2.1%)	2 (0.4%)	0.35
Depth of Submucosal Invasion, ≥ 1000 μm, n (%)					
Absence		124 (23.9%)	124 (23.9%)	0	
Presence		395 (76.1%)	348 (67.1%)	47 (9.1)	< 0.001
Lymphovascular Invasion					
Absence		485 (93.4%)	452 (87.1%)	33 (6.4%)	
Presence		34 (6.6%)	20 (3.9%)	14 (2.7%)	< 0.001
Tumor budding grade (Gr)					
Gr1, low	(0–4 buds/0.785 mm ^2^ )	415 (80%)	388 (74.8%)	27 (5.2%)	
Gr2, intermediate	(5–9 buds/0.785 mm ^2^ )	37 (7.1%)	33 (6.4%)	4 (0.8%)	
Gr3, high	(≥ 10 buds/0.785 mm ^2^ )	67 (12.9%)	51 (9.8%)	16 (3.1%)	< 0.001
Treatment					
Local excision, then colorectal resection		7 (1.3%)	6 (1.2%)	1 (0.2%)	
Primary colorectal resection		512 (98.7)	466 (%)	46 (8.9%)	0.63
AJCC, American Joint Committee on Cancer; CEA, carcinoembryonic antigen; IQR, interquartile range; JSCCR, Japanese Society for Cancer of the Colon and Rectum; LNM, lymph node metastasis; WHO, World Health Organization.					

**Table:** **Table 3** Univariate and multivariate analyses of LNM risk factors.

	**Sensitivity**	**Specificity**	**PPR**	**NPR**	**Univariate**	**Multivariate**	
					***P***	***P***	**RC (95% CI)**
HDG (Gr 3) (WHO 5th)	48.9%	75.2%	16.4%	93.7%	< 0.001	< 0.01	2.87 (1.42–5.78)
LVI (+)	29.2%	95.8%	41.2%	93%	< 0.001	0.03	1.87 (1.06–3.28)
TB Grade 2/3	41.7%	82.2%	19.2%	93.3%	< 0.001	< 0.001	3.58 (1.81–7.09)
DSI (+)	100.0%	26.3%	12.2%	100.0%	< 0.001	0.99	
Gender (female)	57.4%	58.5%	12.1%	93.2%	0.035	< 0.001	2.55 (1.46–4.46)
CI, confidence interval; DSI, deep submucosal invasion; HDG, histological differentiation grade; LN, lymph node; LNM, lymph node metastasis; LVI, lymphovascular invasion; NPR, negative prediction rate; PPR, positive prediction rate; RC, regression coefficient; TB, tumor budding; WHO, World Health Organization.							

Table 4 shows the accuracy of guidelines in predicting LNM based on the unfavorable risk factors they adopt. All guidelines demonstrated significant ability to detect LNM ( *P* < 0.001). NCCN and ESMO had similar performance, in terms of sensitivity (63.8%/68.1%), specificity (62.7%/69.5%), and other parameters. JSCCR and FICPG had similar performance, in terms of good sensitivity (100%/100%), low specificity (25.6%/25.2%), and other parameters. The JSCCR/FICPG group had higher sensitivity, lower specificity and accuracy than the NCCN/ESMO group.

**Table:** **Table 4** Accuracy of guidelines to detect lymph node metastasis.

	**NCCN**	**JSCCR**	**ESMO**	**FICPG**
Sensitivity	63.8%	100.0%	68.1%	100.0%
Specificity	62.7%	25.6%	69.5%	25.2%
Positive prediction rate	14.6%	11.8%	18.2%	11.8%
Negative prediction rate	94.6%	100.0%	95.6%	100.0%
Accuracy	62.8%	32.4%	69.4%	32.0%
ESMO, European Society for Medical Oncology; FICPG, French Intergroup Clinical Practice Guidelines; JSCCR, Japanese Society for Cancer of the Colon and Rectum; NCCN, National Comprehensive Cancer Network.				

## Discussion

HDG, LVI, TB(Gr2/3), and gender are independent risk factors for LNM in multivariate analysis. All guidelines have significant ability to predict LNM. JSCCR/FICPG guidelines have better sensitivity, but lower specificity and accuracy than NCCN/ESMO guidelines. The main reason is because DSI criteria were adopted by JSCCR/FICPG. In this study, DSI is not an independent risk factor, but no tumors with submucosal invasion < 1000 μm had LNM, which is consistent with findings from Japanese studies 4 16 . It is an ideal negative predictor. However, the inclusion of DSI in guidelines would result in a high false-positive ratio, and cause over-recommendation of the need for surgical resection. The low sensitivity of NCCN/ESMO appears to require improvement. Missing one-third of LNM cases is an issue.

Absence of DSI and inclusion of only Grade 3 TB (without Grade 2) are the main differences between NCCN and the other guidelines, which contributes to the low sensitivity of NCCN. However, the significance of DSI (> 1000 μm) remains controversial. A meta-analysis by Zwager et al. suggests that DSI (> 1000 μm, or two-thirds of submucosal invasion) is not an independent risk factor for LNM in T1 cancer 17 . They also reported, in the absence of other high-risk factor, that the ratio of LNM of T1 CRC with only DSI was 2.6%, whereas it was 6.7% (15/224) in this study. How DSI should be applied as an unfavorable risk factor for T1 CRC remains unclear, but excluding DSI as a factor does affect the detection rate and the sensitivity.

In terms of TB, Grade 3 (High) is widely accepted as a significant risk factor for LNM in T1 CRC 2 18 . However, the criteria for applying Grade 2/3 or Grade 3 to determine significance varies. Two major groups—ITBCC 10 and JSCCR 4 —advocate for applying Grade 2/3 TB criteria. The reported incidence of positive TB in T1 CRC ranges from 11.8% to 75%, depending on the criteria, tissue staining, or diagnostic methods used 19 20 21 . Typically, it falls between 10% and 20%, and our Grade 2/3 incidence (20%) is consistent with other reports.

While the NCCN guideline low sensitivity may seem concerning, it is important to note that the algorithm includes an indefinite decision (observation or colectomy) for sessile T1 colon cancers without unfavorable factors 5 . Its approach reminds clinicians that sessile lesions have a significantly higher incidence of adverse outcomes. However, our analysis was limited to cases with well-defined unfavorable factors and could not include the indefinite decision model. In addition, pedunculated T1 tumors without risk factors are not free of LNM, albeit with a very low incidence 18 .

The major concern regarding these unfavorable risk factors is interobserver variability, particularly for DSI. In 2004, Ueno et al. introduced the SMID measurement method, which was later adopted by JSCCR. This includes two types of depth measurements 20 . The first uses muscularis mucosae (MM) as the upper reference line (Y1), whereas the second uses tumor surface (Y2) when MM is not recognized. The primary issue is that estimation of MM is observer-dependent, leading to variability even after training 22 . There is also the pathologist-dependent variation about Y1 or Y2 criteria usage. To minimize variability, multiple pathologists should be involved in reviewing pathological parameters. One of this study’s major limitation is that only one experienced pathologist conducted the review. We suggest that a study be conducted to verify the impact of these two measurement methods.

A comparison of validation results among relevant series is shown in Table 5 23 24 25 . The good sensitivity of JSCCR is confirmed in all studies from different areas. The reported sensitivity of NCCN ranges from 63.8% to 100%; ESMO is 68.1% to 100%, and our series is the lowest. In terms of specificity, NCCN is 44% to 82.4%, JSCCR 0% to 25.6%, and ESMO 0% to 69.5%. It is surprising that Ichimasa et al reported 0% specificity on JSCCR/ESMO 26 . We think our low LVI incidence is the main reason for the low sensitivity and high specificity of NCCN/ESMO. The incidence of LVI (6.6%) in this study is lower than previously reported (10% to 21%) 27 28 . Bosch et al. reported 14.1% of LVI in their meta-analyses of 17 studies 2 . It is hard to explain our lower LVI rate, given to the same adopted criteria for LVI diagnosis. This lower LVI rate can probably be explained by the more conservative attitude of our pathologist in reaching LVI diagnosis. However, on reviewing the LVI rate in our whole CRC database, we found a reasonably progressive increasing LVI rate according to increasing staging. Special staining labelling lymphatic vessels can increase LVI detection. On the basis of confirmed LVI impact on LNM risk of T1 CRC, a comparative study of H&E and lymphatic vessel staining is recommended.

**Table:** **Table 5** Sensitivity and specificity of reported validation studies.

**Study, Year**			**NCCN**	**JSCCR**	**ESMO**		**NCCN**	**JSCCR**	**ESMO**
Ichimasa et al. 23	2018 (n = 690)	Sensitivity	100%	100%	100%	Specificity	44%	0	0
Piao et al. 24	2023 (n = 651)		87.5%	100%	NA		82.4%	17.5%	NA
Tanino et al. 25	2024 (n = 560)		98%	100%	98%		52%	19%	50%
This study	(n = 519)		63.8%	100%	68.1%		62.7%	25.6%	69.5%
ESMO, European Society for Medical Oncology; JSCCR, Japanese Society for Cancer of the Colon and Rectum; NA, not available; NCCN, National Comprehensive Cancer Network.									

The HDG evaluation criteria differ significantly between JSCCR and NCCN/ESMO. In JSCCR, it is defined as the predominant histological grade according to their guidelines 9 . JSCCR HDG criteria did not correlate with independent significance with LNM in either study. The HDG criteria for NCCN/ESMO are based on the WHO classification of tumors (5th) 13 : Presence of the least or poorly differentiated pathology. Prevalence of this HDG definition in this study is similar to the report by Tanino et al (27% vs. 20%). It correlates with LNM in the univariate and multivariate analyses in both studies.

This study is based on the assumption that all T1 CRCs are well excised, fixed, and examined. However, this is not always the case, because factors such as tumor size, morphology, location, and procedure execution can affect outcomes. In our study, most specimens from initial LE cases were not suitable for review, which led to their exclusion. However, in terms of current clinical procedure, pathologies according to endoscopic excision first are common. This selection bias should be acknowledged in this study. This illustrates how diverse results can be, even with well-defined pathological factors. To ensure precise pathological interpretation for further decisions, a standardized procedure should be implemented.

Physicians must carefully consider both the benefits and risks of surgery when assessing LNM risk in T1 CRC. Table 6 shows related results of previously published studies 1 29 30 31 32 33 34 35 . Regarding surgical mortality for specific T1 CRC series, there were few previous reports. Two studies 30 32 reported no surgical mortality; Belderbos et al. 31 reported a little higher 30-day mortality of 2.6%, whereas there is only case (1.9‰) in our study. The recurrence rate after curative resection, including primary surgical resection or additional resection after LE, was 1.9% to 4.3%, seemingly related to the LNM+ rate in each study except that of Belderbos et al. In our study, during a medium follow-up of 5.44 years (IQR, 2.45 to 8.23), there were 10 distant recurrences, without any local recurrence, and five of the patients died of CRC. With treatments according to JSCCR/FICPG guidelines, approximately 90% of resections will be over-done, although with very low surgical mortality rate. If with NCCN/ESMO guidelines, 81.9% to 85.4% of resections will be over-done, but will miss one-third of LNM cases. More validation studies of guidelines will be needed to examine these relevant results. Generally speaking, surgical resection risk is low. If LNM risk can be predicted more accurately, the benefit of surgery will be enhanced. Besides oncological outcomes, surgeons’ other concern is functional outcomes after rectal surgery, such as low anterior resection syndrome (LARS), which should be also considered and explained in detail to patients.

**Table:** **Table 6** LNM rate, surgical mortality, and recurrence rate of reported series of T1 colorectal cancers treated with curative resection.

**Study, Year**		**Follow-up (year)**	**LNM+ n (%)**	**Surgical mortality rate**	**Recurrence, n (%)**
Kobayashi et al. 29	2011 (n = 798)	Median (range), 1.9 (0.3–4.4)	84 (10.5%)	NA	18 (2.3%)
Yoda et al. 30	2013 (n = 202)	Median (range), 5 (1–5.25)	23 (11.4%)	0	7 (3.5%)
Belderbos et al. 31	2017 (n = 945)	Median, 6.6	82 (8.7%)	25 (2.6%)	39 (4.1%)
Liu et al. 32	2020 (n = 481)	NA	55 (11.4%)	0	14 (2.9%)
Chang et al. 2	2021 (n = 318)	Mean ± SD, 6.8 ± 2.62	50 (15.7%)	NA	11 (3.5%)
Ha et al. 33	2022 (n = 466)	Median (IQR), 6 (4.92–8.08)	NA	NA	9 (1.9%)
Tamaru et al. 34	2024 (n = 2438)	Mean, 5.53	226 (9.3%)	NA	88 (3.6%)
Nilsson et al. 35	2024 (n = 1317)	Median (IQR), 5 (3.17–5.29)	154 (11.7%)	NA	48 (3.6%)
This study	(n = 519)	Medium (IQR), 5.44 (2.45–8.23)	47 (9.1%)	1 (1.9‰)	10 (1.9%)
IQR, interquartile range; LNM, lymph node metastasis; NA, not available; SD, standard deviation.					

There are limitations to this study. This was a retrospective, single-center series, and many pathological materials were excluded due to improper preparation, particularly from LE first cases. Occasionally, faded slide staining was not suitable for review, even after re-preparation from preserved paraffin blocks. This could affect accuracy of pathology. In addition, review of endoscopic morphology was limited, and final pathological and clinical reviews did not reach the initially designed sample size, reducing the study power. Selection and background biases including the fact that most LE specimens were not suitable for analyses, as well as use of a single pathologist, also limit study validity. Finally, differing algorithms used in the guidelines make direct comparisons challenging.

## Conclusions

In conclusion, HDG, LVI, TB(Gr2/3), and gender are independent risk factors for LNM of T1 CRC. DSI is an excellent negative predictor although not an independent risk factor. NCCN/ESMO guidelines have medium sensitivity and require improvement. JSCCR/FICPG has perfect sensitivity but low specificity, thus exposing patients to many unnecessary surgeries. There is potential for revision of current guideline factors, including those beyond current pathological ones, to improve LNM prediction accuracy.
